# Author Correction: *O*-GlcNAc modification of leucyl-tRNA synthetase 1 integrates leucine and glucose availability to regulate mTORC1 and the metabolic fate of leucine

**DOI:** 10.1038/s41467-022-34146-3

**Published:** 2022-10-21

**Authors:** Kibum Kim, Hee Chan Yoo, Byung Gyu Kim, Sulhee Kim, Yulseung Sung, Ina Yoon, Ya Chun Yu, Seung Joon Park, Jong Hyun Kim, Kyungjae Myung, Kwang Yeon Hwang, Sunghoon Kim, Jung Min Han

**Affiliations:** 1 Interdisciplinary Program of Integrated OMICS for Biomedical Science, Graduate School, Yonsei University, Seoul, 03722 South Korea; 2 Yonsei Institute of Pharmaceutical Sciences, College of Pharmacy, Yonsei University, Incheon, 21983 South Korea; 3 Center for Genomic Integrity, Institute for Basic Science, Ulsan, 44919 South Korea; 4 Department of Biotechnology, College of Life Sciences and Biotechnology, Korea University, Seoul, 02841 South Korea; 5 Institute for Artificial Intelligence and Biomedical Research, Medicinal Bioconvergence Research Center, Yonsei University, Incheon, 21983 South Korea; 6 College of Medicine, Gangnam Severance Hospital, Yonsei University, Seoul, 06273 South Korea; 7 Department of Biochemistry, School of Medicine, Catholic University of Daegu, Daegu, 42472 South Korea; 8 Department of Biomedical Engineering, Ulsan National Institute of Science and Technology, Ulsan, 44919 South Korea; 9 POSTECH Biotech Center, Pohang University of Science and Technology, Pohang, 37673 South Korea

**Keywords:** Nutrient signalling, Glycobiology, Post-translational modifications, Metabolism

Correction to: *Nature Communications* 10.1038/s41467-022-30696-8, published online 25 May 2022

The original version of this Article contained an error in the top panel of Fig. 3d and the corresponding source data file, in which a wrong immunoblot image was shown. This has been corrected in both the PDF and HTML versions of the Article.

