# Supplementary material for: O-GlcNAc modification of leucyl-tRNA synthetase 1 integrates leucine and glucose availability to regulate mTORC1 and the metabolic fate of leucine
Source: Nat Commun. 2022 May 25;13:2904. doi: 10.1038/s41467-022-30696-8 (PMC9133088; doi:10.1038/s41467-022-30696-8)
Supplement: Supplementary file 1 — Supplementary Information [file 41467_2022_30696_MOESM1_ESM.pdf]

Supplementary information

O-GlcNAc modification of leucyl-tRNA synthetase 1 integrates leucine and glucose availability to regulate mTORC1 and the metabolic fate of leucine

Kibum Kim[1,2,10], Hee Chan Yoo[2,10], Byung Gyu Kim[3], Sulhee Kim[4], Yulseung Sung[2], Ina Yoon[2,5,6], Ya Chun Yu[2], Seung Joon Park[1,2], Jong Hyun Kim[7], Kyungjae Myung[3,8], Kwang Yeon Hwang[4], Sunghoon Kim[2,5 6], Jung Min Han[1,2,9,*]

Affiliations

1. Interdisciplinary Program of Integrated OMICS for Biomedical Science, Graduate School, Yonsei University, Seoul 03722, South Korea
2. Yonsei Institute of Pharmaceutical Sciences, College of Pharmacy, Yonsei University, Incheon 21983, South Korea
3. Center for Genomic Integrity, Institute for Basic Science, Ulsan 44919, South Korea
4. Department of Biotechnology, College of Life Sciences and Biotechnology, Korea University, Seoul 02841, South Korea
5. Institute for Artificial Intelligence and Biomedical Research, Medicinal Bioconvergence Research Center, Yonsei University, Incheon 21983, South Korea
6. College of Medicine, Gangnam Severance Hospital, Yonsei University, Seoul 06273, South Korea
7. Department of Biochemistry, School of Medicine, Catholic University of Daegu, Daegu 42472, South Korea.
8. Department of Biomedical Engineering, Ulsan National Institute of Science and Technology, Ulsan 44919, South Korea
9. POSTECH Biotech Center, Pohang University of Science and Technology, Pohang 37673, South Korea
10. These authors contributed equally

*Correspondance: jhan74@yonsei.ac.kr (J.M.H.)

This PDF file includes :

Supplementary Figures 1-8 and Table 1-2

Supplementary Fig. 1

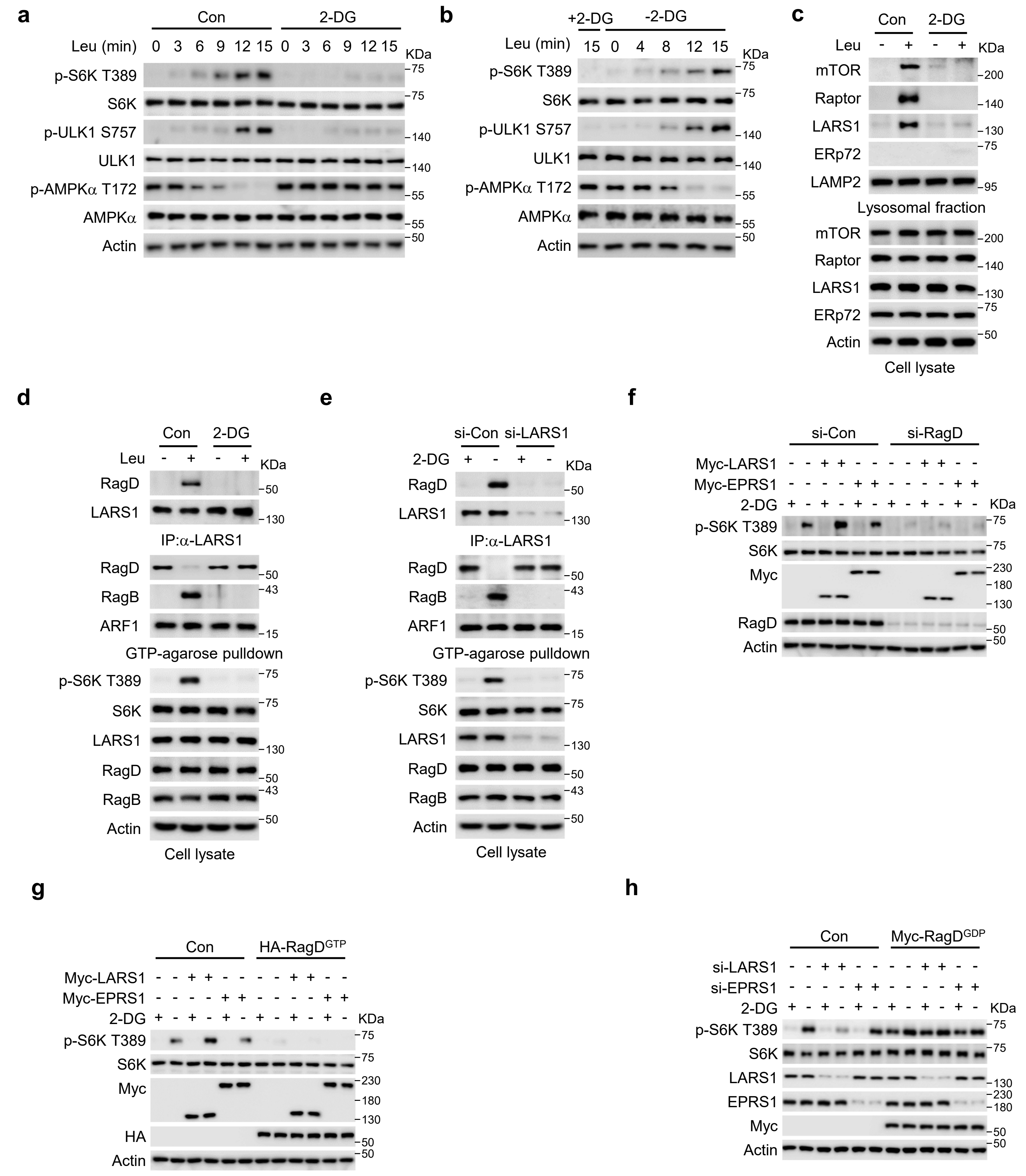

Supplementary Fig. 1. Inhibitory Effect of 2-deoxy-d-glucose (2-DG) on the LARS1-mTORC1 axis.

a SW620 cells were starved of leucine for 1.5 h with or without 100 mM 2-DG and stimulated with 0.4mM leucine for the indicated durations. **b** For the 2-DG sample, cells were starved of leucine for 1.5 h and stimulated with 0.4 mM leucine and 100 mM 2-DG for 15 min. For samples in which 2-DG was removed, cells were starved of leucine for 1.5 h and stimulated with 0.4 mM leucine and 100 mM 2-DG for 15 min. After 15 min of stimulation, the culture medium was exchanged with fresh medium containing 0.4 mM leucine without 2-DG for the indicated durations. **a**, **b** The cells were harvested, and each cell lysate was immunoblotted with the indicated antibodies. **c**, **d** SW620 cells were starved of leucine for 1.5 h and stimulated with 0.4mM leucine for 15 min with or without 100 mM 2-DG. **e** SW620 cells were transfected with control or siRNA against LARS1. After 48 h, the cells were incubated with or without 100 mM 2-DG for 15 min. **c** Each cell lysate was used for lysosomal fractionation and immunoblotted with the indicated antibodies. **d**, **e** Each cell lysate was precipitated with anti-LARS1 antibody-conjugated agarose beads or GTP-conjugated agarose beads and analyzed by immunoblotting with the indicated antibodies. **f**, **g**, **h** SW620 cells were transfected with the indicated expression constructs or siRNAs. After constructs or siRNAs transfection, the cells were incubated with or without 100 mM 2-DG for 15 min. Each cell lysate was analyzed by immunoblotting with the indicated antibodies. Representative data of three experiments with similar results. Source data are provided as a Source Data file.

Supplementary Fig. 2

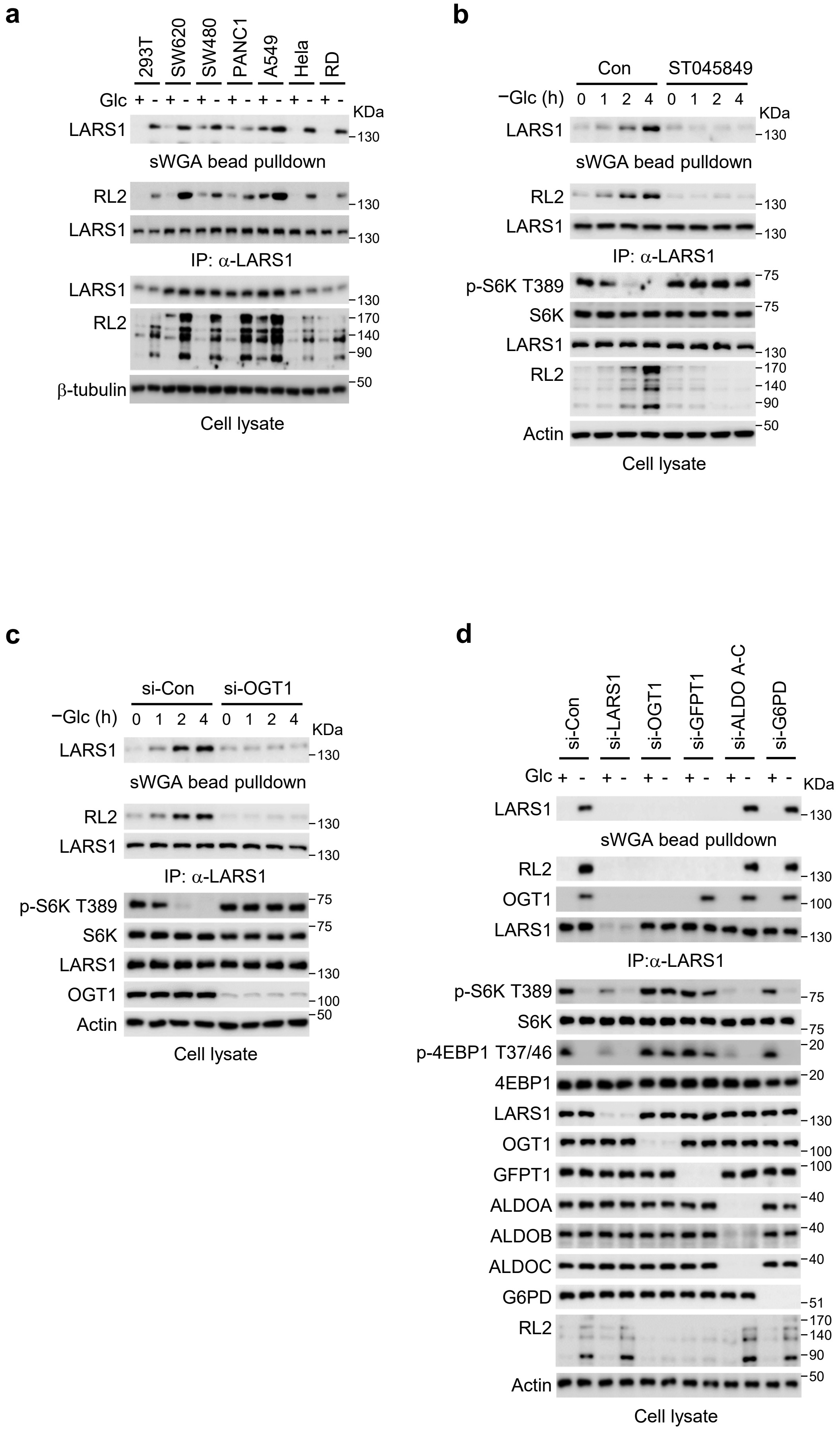

Supplementary Fig. 2. glucose starvation induced LARS1 *O*-GlcNAcylation through OGT1.

a Each indicated cell sample was incubated with or without glucose for 4 h (25mM glucose for 293T, PANC1, HeLa, and RD cells, or 11mM glucose for SW620, SW480, and A549 cells). **b** SW620 cells were incubated with vehicle or 20 μM ST045849. After 24 h, the cells were starved of glucose for the indicated durations. **c** SW620 cells were transfected with control or siRNA against OGT1. After 48 h, the cells were starved of glucose for the indicated durations. **d** SW620 cells were transfected with the indicated siRNAs. After 48 h, the cells were starved of glucose for 4 h and supplemented with glucose for 30 min. **a**, **b**, **c**, **d** Each cell lysate was precipitated with sWGA-conjugated agarose beads or anti-LARS1 antibody-conjugated agarose beads and analyzed by immunoblotting with the indicated antibodies. Representative data of three experiments with similar results. Source data are provided as a Source Data file.

Supplementary Fig. 3

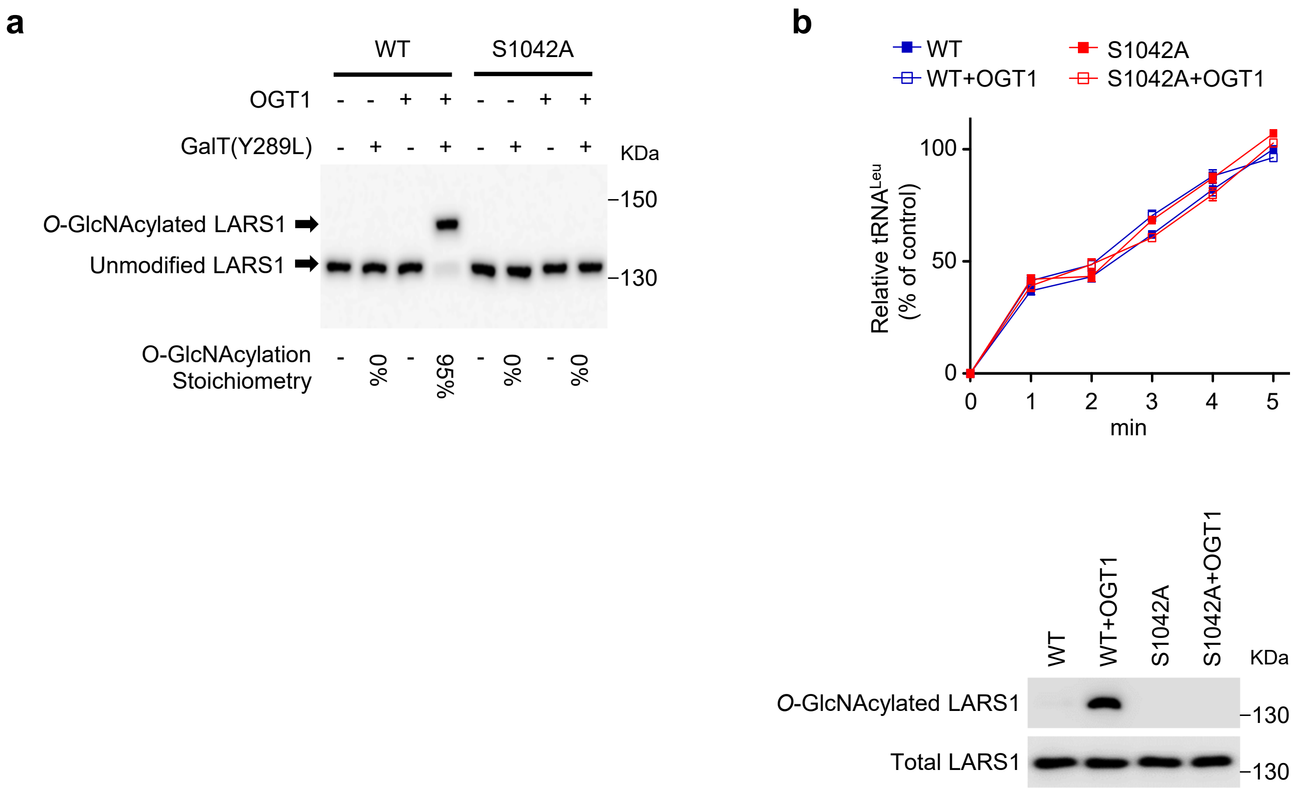

Supplementary Fig. 3. LARS1 *O*-GlcNAcylation does not influence leucylation capacity of LARS1

a, b His-tagged WT and S1042A mutant LARS1 were purified from *E. coli* with or without WT or N567K OGT1 expression. **a** GalTY289L labeling of *in vitro* *O*-GlcNAcylated LARS1 WT and S1042A LARS1 analyzed by immunoblotting with LARS1 antibody. **b** Each LARS1 protein was incubated with 4mM ATP, 2mg/ml tRNA, 1μM [^{3}H] Leucine for LARS1 aminoacylation assay. Inset, LARS1 proteins were expressed with or without OGT1 from *E.coli*. LARS1 proteins were purified with Ni-NTA beads, precipitated with sWGA-conjugated agarose beads and analyzed by immunoblotting with LARS1 antibody. Mean±SEM, n=3 independent experiments. Representative data of three experiments with similar results. Source data are provided as a Source Data file.

Supplementary Fig. 4

a

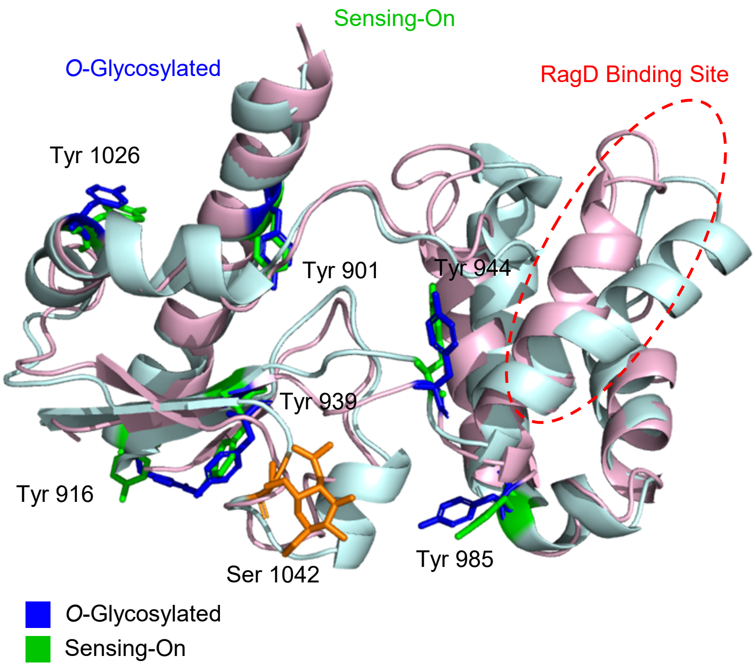

b

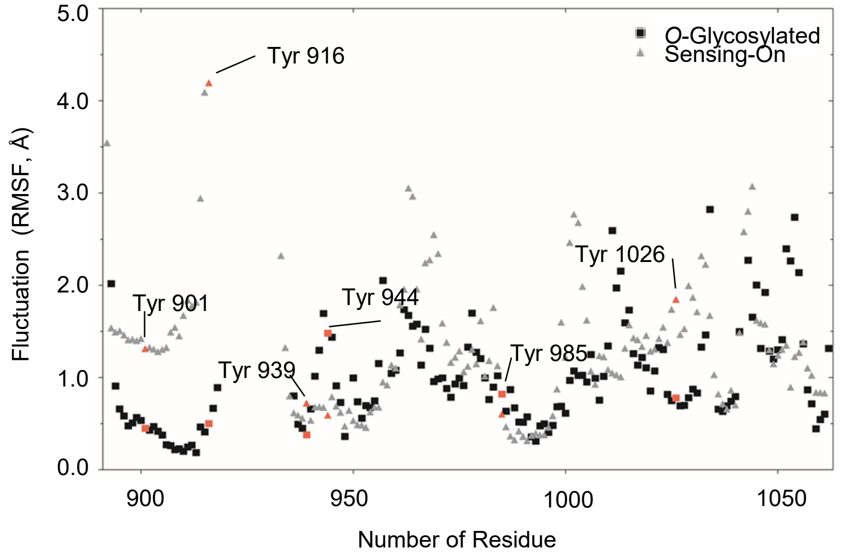

c

Homo sapiens	NEVLIHSSQYLMEVTHDLRLRLKNYMMPAKGKKTDKQ	929
Rhinopithecus bieti	DEVLIHSSQYLMEVTHDLRLRLKNYMMPAKGKKTDKQ	916
Gorilla gorilla	DEVLIHSSQYLMEVTHDLRLRLKNYMIPAKGKKTDKQ	930
Nomascus leucogenys	DEVLIHSSQYLMEVTHDLRLRLKNYMMPAKGKKTDKQ	924
Homo sapiens	PLQKPSHCTIYVAKNYPPWQHTTLSVLRKHFEANNGK	966
Rhinopithecus bieti	PLQKPSHCTIYVAKNYPPWQHTTLSVLRKHFEANNGK	953
Gorilla gorilla	PLQKPSHCTIYVAKNYPPWQHTTLSVLRKHFEANNGK	967
Nomascus leucogenys	PLQKPSHCTIYVAKNYPPWQHTTLSVLRKHFEANNGK	961
Homo sapiens	LPDNKVIASELGSMPELKKYMKKVMPFVAMIKENLEK	1003
Rhinopithecus bieti	LPDNKVIASELGSMPELKKYMKKVMPFVAMIKENLEK	990
Gorilla gorilla	LPDNKVIASELGSMPELKKYMKKVMPFVAMIKENLEK	1004
Nomascus leucogenys	LPDNKVIASELGSMPELKKYMKKVMPFVAMIKENLEK	998
Homo sapiens	MGPRILDLQLEFDEKAVLMENIVYLTNSLELEHIEVK	1040
Rhinopithecus bieti	MGPRILDLQLEFDEKAVLMENIVYLTNSLELEHIEVK	1027
Gorilla gorilla	MGPRILDLQLEFDEKAVLMENIVYLTNSLELEHIEVK	1041
Nomascus leucogenys	MGPRILDLQLEFDEKAVLMENIVYLTNSLELEHIEVK	1035
Homo sapiens	FASEAEDKIREDCCPGKPLNVFRIEPGVSVSLVNPQP	1077
Rhinopithecus bieti	FASEAEDKIREDCCPGKPLNVFRIEPGVSVSLVNPQP	1064
Gorilla gorilla	FASEVEDKIREDCCPGKPLNVFRIEPGVSVSLVNPQP	1078
Nomascus leucogenys	FASEAEDKIREDCCPGKPLNVFRIEPGVSVSLVNPQP	1072

Supplementary Fig. 4. Structural change of LARS1 VC domain by S1042 *O*-GlcNAcylation.

a The structures of the VC domains of *O*-GlcNAcylated (colored cyan) and “sensing-on” LARS1 (colored pink) were superimposed. Each RagD-binding site is highlighted by a dotted line, and the locations of tyrosine residues are colored blue for the *O*-GlcNAcylated structure and green for the “sensing-on” structure. *O*-GlcNAcylated S1042 is colored orange. **b** Structural flexibility profile showing fluctuations in amino acid residues within the VC domain of LARS1 from the “sensing-on” and *O*-GlcNAcylated structures. The fluctuation score of amino acids from the “sensing-on” structure is marked as a gray triangle, and that of the *O*-GlcNAcylated structure is marked as a black square. Tyrosine residues are colored orange. RMSF; root mean square fluctuation. **c** An amino acid sequence alignment of the VC domains of LARS1 from humans, *Rhinopithecus bieti*, *Gorilla gorilla*, and *Nomascus leucogenys* shows the locations of conserved tyrosine residues marked in red.

Supplementary Fig. 5

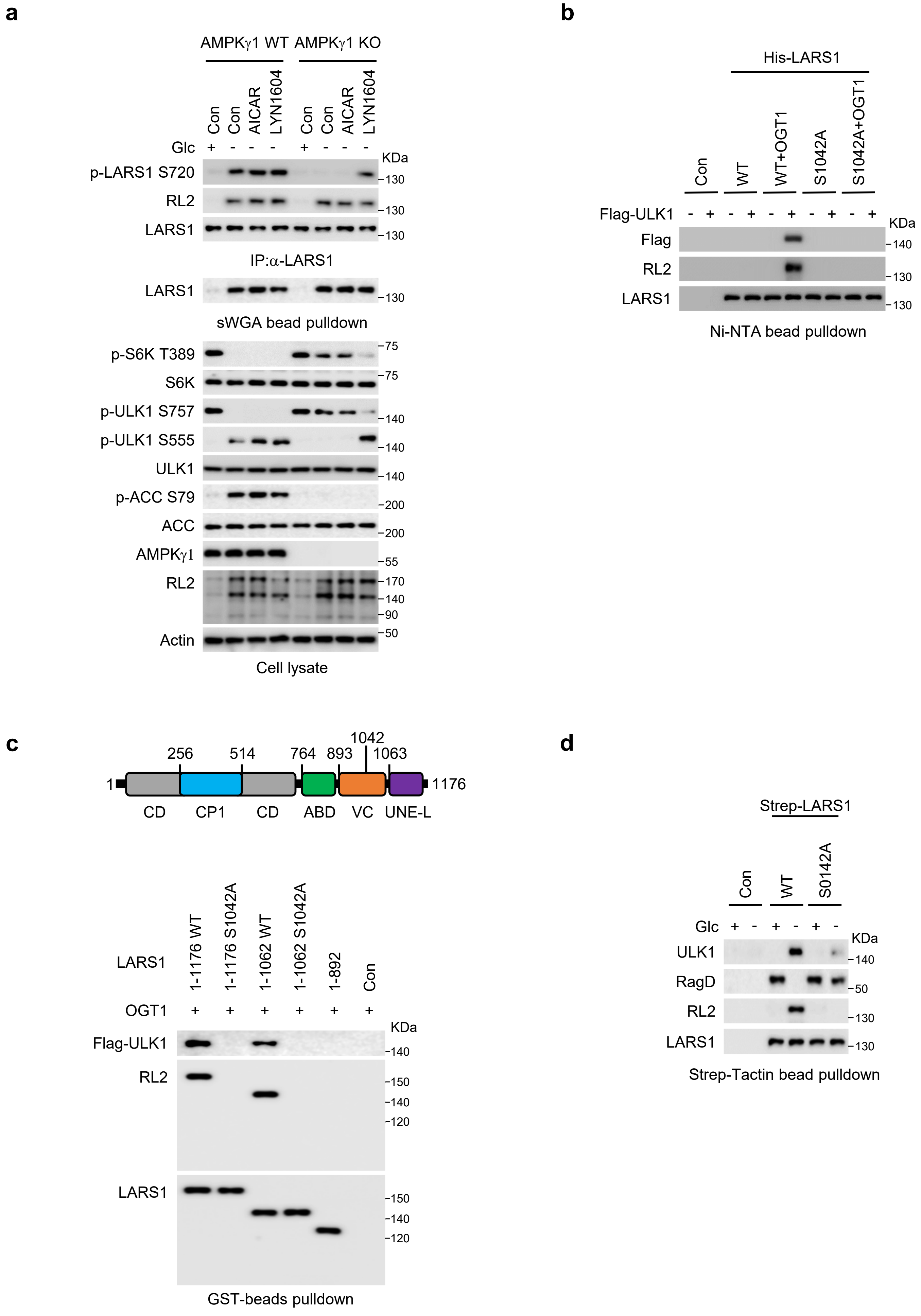

Supplementary Fig. 5. The *O*-GlcNAcylation of LARS1 controls ULK1 binding.

a AMPKγ1 WT or KO 293A cells were starved of glucose for 4 h with vehicle or indicated compounds (1μM LYN1604, 1mM AICAR). Each sample was subjected to immunoprecipitation with sWGA-conjugated agarose beads, anti-LARS1 antibody-conjugated beads or anti-myc antibody-conjugated agarose beads and analyzed by immunoblotting with the indicated antibodies. **b** His-tagged WT LARS1 and S1042A mutant LARS1 were purified from *E. coli* with or without OGT1 expression. Each protein sample was incubated with cell lysate with or without Flag-ULK1 protein overexpression. After 1 h of incubation, immunoblotting was performed using the indicated antibodies, and proteins were stained with Coomassie blue. **c** GST-tagged LARS1 proteins were purified form *E.coli* with OGT1 expression. Each protein sample was incubated with cell lysates expressing Flag-tagged ULK1. After 1 h of incubation, immunoblotting was performed using the indicated antibodies. **d** SW620 cells were transfected with the indicated strep-tagged LARS1 constructs. After 24 h, the cells were starved of glucose for 4 h and supplemented with 11mM glucose for 30 min. Then, each cell sample was harvested, and strep-tagged WT LARS1 and S1042A mutant LARS1 were precipitated with Strep-Tactin Sepharose beads. Each sample was immunoblotted with the indicated antibodies. Representative data of three experiments with similar results. Source data are provided as a Source Data file.

Supplementary Fig. 6

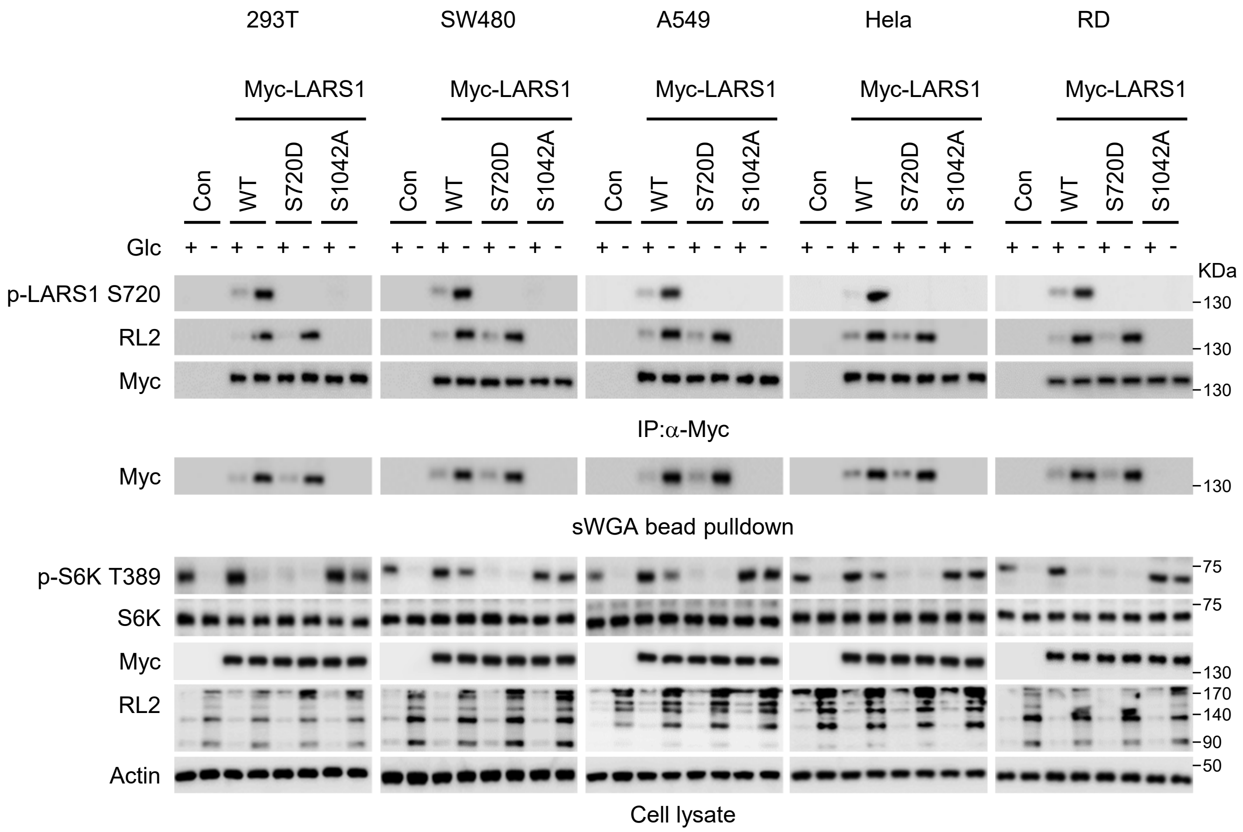

Supplementary Fig. 6. Effect of glucose availability on the *O*-GlcNAcylation and phosphorylation of LARS1 in various cell lines.

Each indicated cell line was transfected with the indicated LARS1 constructs. After 24 h, each cell line was starved of glucose for 4 h and supplemented with glucose for 30 min (25mM glucose for 293T, HeLa, and RD cells, or 11mM glucose for SW620, SW480, and A549 cells). Each cell lysate was precipitated with sWGA-conjugated agarose beads or anti-LARS1 antibody-conjugated agarose beads and analyzed by immunoblotting with the indicated antibodies. Representative data of three experiments with similar results. Source data are provided as a Source Data file.

Supplementary Fig. 7

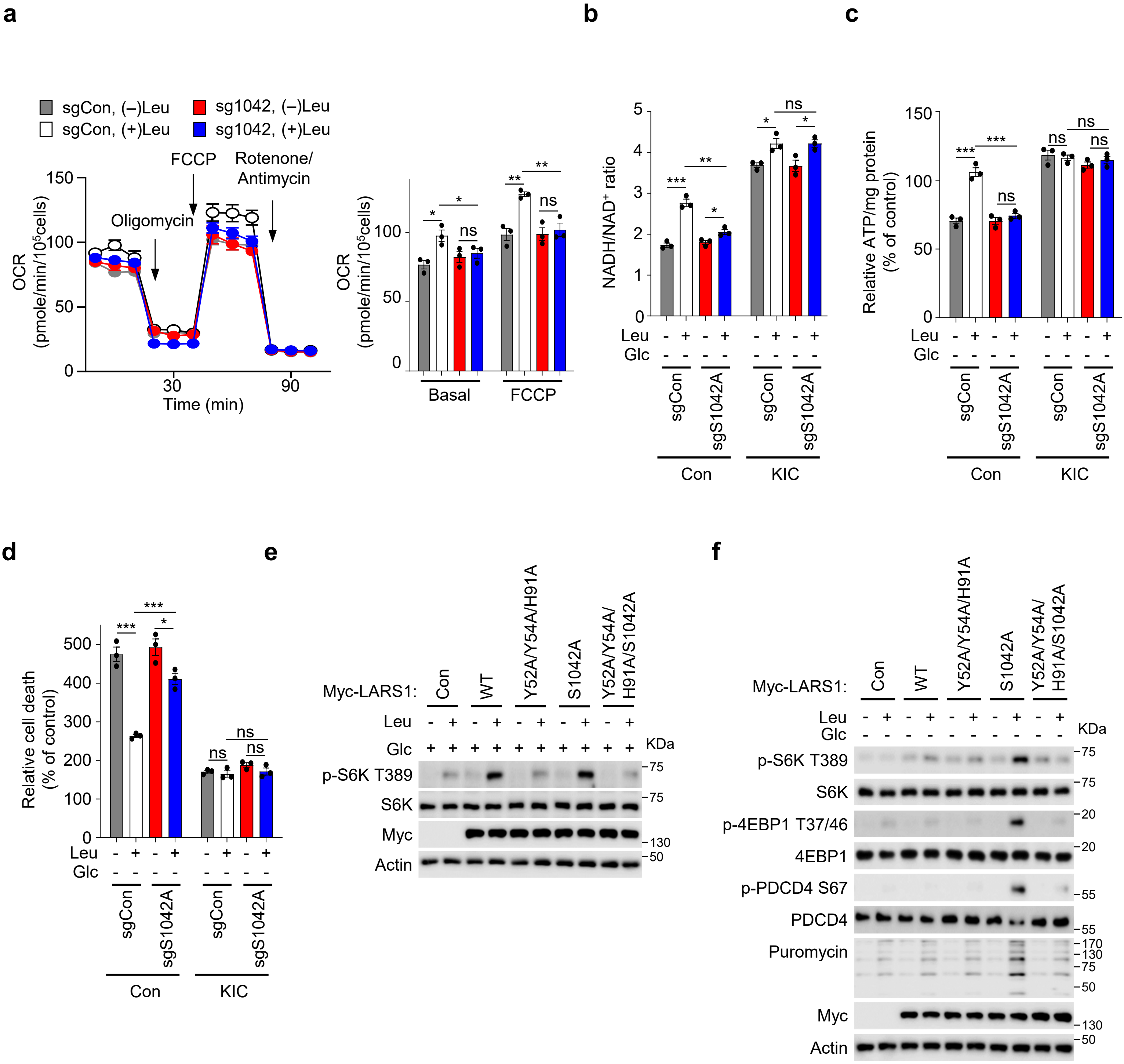

Supplementary Fig. 7. The *O*-GlcNAcylation of LARS1 controls leucine-derived ATP production and leucine-induced mTORC1 activation.

a, **b**, **c**, **d** SW620 control or S1042A knock-in cells were detached and seeded in 24-well plates, starved of glucose and leucine for 4 h then leucine was added for 4 h. **a** the cells were exposed to 2 μM oligomycin, 0.5 μM FCCP, and 0.5 μM/0.5 μM rotenone/antimycin, and the OCR was measured. Left: OCR over time; right: bar graph of basal OCR and FCCP-treated maximal OCR from left (mean ±SEM, n=3, independent experiments). For the measurement of intracellular ATP, cells were harvested and analyzed with **b** NADH/NAD^+ assay kit, **c** ATP assay kit. **d** For monitoring cell death, cells were incubated with CellTox™ Green dye and the green fluorescence signals from dead cells were detected by a live-cell imaging analyzer (mean±SEM, n=3, independent experiments). **e**, **f** SW620 cells were transfected with the indicated LARS1 constructs; after 24 h of transfection, **e** the cells were starved of leucine for 1.5 h and stimulated with 0.4mM leucine for 15 min. **f** The cells starved of glucose and leucine for 4 h then leucine was added for 4 h. The cells were then harvested, and each cell lysate was immunoblotted with the indicated. *P*-value was determined by two-tailed unpaired Student's *t* test. *, $P<0.05$; **, $P<0.01$; ***, $P<0.001$; ns, not significant. Representative data of three experiments with similar results. Source data are provided as a Source Data file.

Supplementary Fig. 8

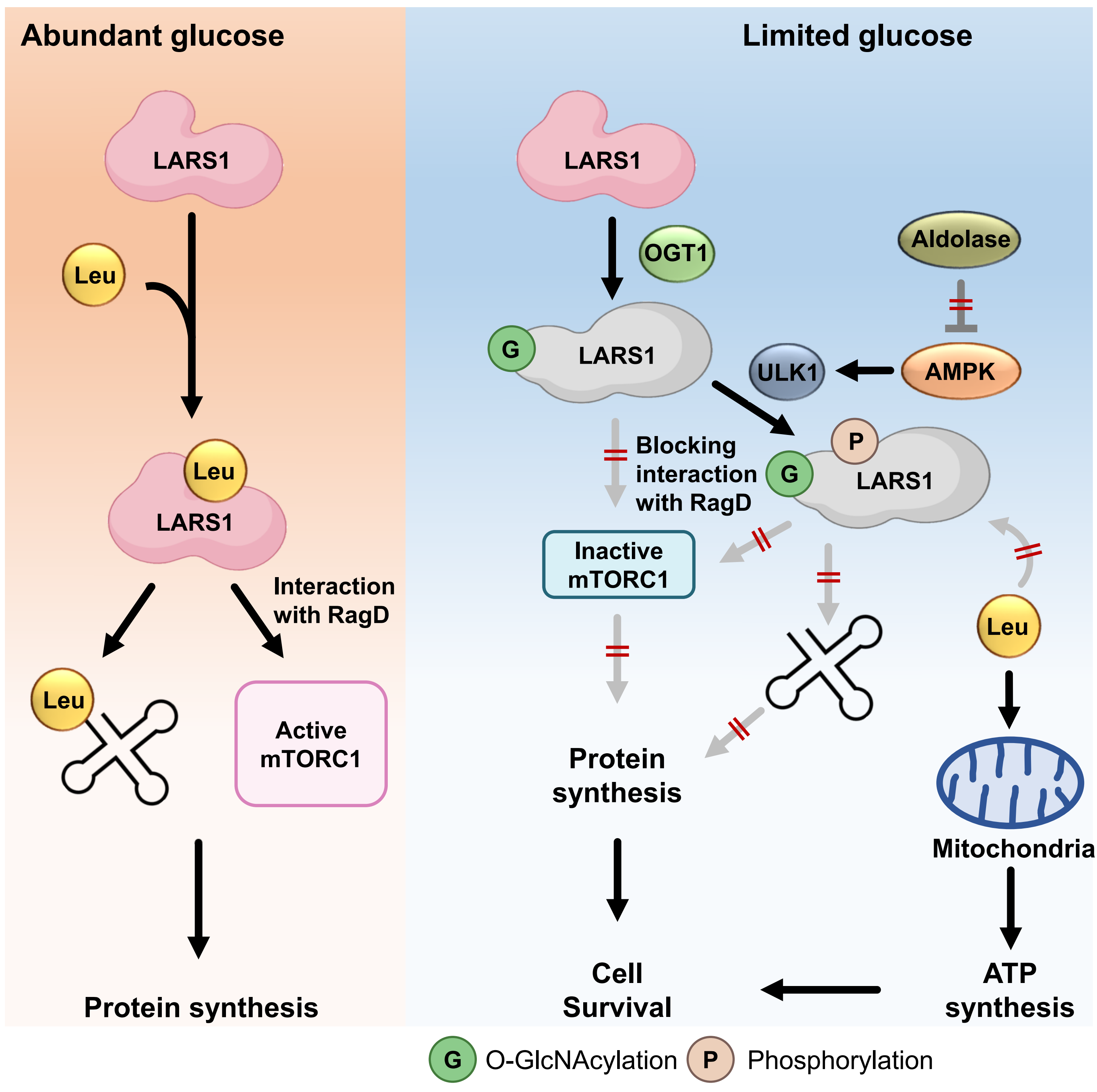

Supplementary Fig. 8. Glucose availability controls LARS1-mTORC1 axis via *O*-GlcNAcylation

In abundance of glucose, LARS1 participates not only in the synthesis of leucyl-tRNA but also the activation of mTORC1 signaling, resulting in enhanced protein synthesis in cells. Conversely, under the circumstance of limited glucose, LARS1 is *O*-GlcNAcylated at serine 1042 by OGT1 and subsequently phosphorylated at serine 720 by ULK1. These post-translational modifications of LARS1 consequently suppress mTORC1 signaling. Leucine not participated in protein synthesis is catabolized for generating ATP and protecting cells from death.

Supplementary Table 1. Sequences of siRNA

Human AMPKa siRNA	5'-AGGAGAGCUAUUUGAUUA-3'
Human AMPKg siRNA	5'-GGTGGACATCTACTCCAAGTT-3'
Human LARS1 siRNA	5'-CCAGGGUCAUUGUCGUGGAUUUGCA-3'
Human OGT1 siRNA	5'-GAUUAAGCCUGUUGAAGUC-3',<br>5'-GCUUGCAAUUCAUCACUUU-3'
Human GFPT1 siRNA	5'-CAGAGGAUAUGAUUCUGCUGGUGU-3'
Human ULK1 siRNA	5'-GUGGCCCUGUACGACUUC-3',<br>5'-UUUCCUGGAAGUCGUACA-3'
Human ULK2 siRNA	5'-CCAAAAAGAUAGAAUGGA-3',<br>5'-UUCAAAGUCCAUUCUAUC-3'

Supplementary Table 2. For LARS1 point mutation, sequences of primer

LARS1 Y52A/Y54A	F	5’-GTAACCTTCCCAGCTCCAGCCATGAATGGACGCCTTCATTTGG-3’
	R	5’-CCAAATGAAGGCGTCCATTCATGGCTGGAGCTGGGAAGGTTAC-3’
LARS1 H91A	F	5’-TCCCTTTGGCCTGGCCTGTACTGGAATGCCTATTAAGG-3’
	R	5’-CCTTAATAGGCATTCCAGTACAGGCCAGGCCAAAGGGA-3’
LARS1 S621A	F	5’-TTGCATGGACAGGCAGAGGCTCCGCTGGGCATTAGAC-3’
	R	5’-GTCTAATGCCCAGCGGAGCCTCTGCCTGTCCATGCAA-3’
LARS1 S720A	F	5’- CTGAACTCTGAGAAGATGTCAAAAGCCACAGGCAACTT-3’
	R	5’-AAGTTGCCTGTGGCTTTTGACATCTTCTCAGAGTTCAG-3’
LARS1 S720D	F	5’-CTGAACTCTGAGAAGATGTCAAAAGACACAGGCAACTT-3’
	R	5’-AAGTTGCCTGTGTCTTTTGACATCTTCTCAGAGTTCAG-3’
LARS1 T728A	F	5’-CACAGGCAACTTCCTCACTTTGGCCCAAGCTATTGACAAATTTTC-3’
	R	5’-GAAAATTTGTCAATAGCTTGGGCCAAAGTGAGGAAGTTGCCTGTG-3’
LARS1 S790A	F	5’-CAGCCTAAGAAGTGGTCCTGCCGCCACTTTCAATGATAGAGTTTTTG-3’
	R	5’-CAAAAACTCTATCATTGAAAGTGGCGGCAGGACCACTTCTTAGGCTG-3’
LARS1 S1042A	F	5’-CACATAGAAGTCAAGTTTGCCGCCGAAGCAGAAGATAAAAT-3’
	R	5’-ATTTTATCTTCTGCTTCGGCGGCAAACTTGACTTCTATGTG-3’
