# Supplementary material for: O-GlcNAc modification of leucyl-tRNA synthetase 1 integrates leucine and glucose availability to regulate mTORC1 and the metabolic fate of leucine
Source: Nat Commun. 2022 May 25;13:2904. doi: 10.1038/s41467-022-30696-8 (PMC9133088; doi:10.1038/s41467-022-30696-8)
Supplement: Supplementary file 3 — Reporting Summary [file 41467_2022_30696_MOESM3_ESM.pdf]

nature portfolio

Corresponding author(s): Jung Min Han

Last updated by author(s): May 2, 2022

Reporting Summary

Nature Portfolio wishes to improve the reproducibility of the work that we publish. This form provides structure for consistency and transparency in reporting. For further information on Nature Portfolio policies, see our Editorial Policies and the Editorial Policy Checklist.

Statistics

For all statistical analyses, confirm that the following items are present in the figure legend, table legend, main text, or Methods section.

n/a	Confirmed	
☐	☒	The exact sample size (*n*) for each experimental group/condition, given as a discrete number and unit of measurement
☐	☒	A statement on whether measurements were taken from distinct samples or whether the same sample was measured repeatedly
☐	☒	The statistical test(s) used AND whether they are one- or two-sided<br>*Only common tests should be described solely by name; describe more complex techniques in the Methods section.*
☒	☐	A description of all covariates tested
☒	☐	A description of any assumptions or corrections, such as tests of normality and adjustment for multiple comparisons
☐	☒	A full description of the statistical parameters including central tendency (e.g. means) or other basic estimates (e.g. regression coefficient) AND variation (e.g. standard deviation) or associated estimates of uncertainty (e.g. confidence intervals)
☐	☒	For null hypothesis testing, the test statistic (e.g. *F*, *t*, *r*) with confidence intervals, effect sizes, degrees of freedom and *P* value noted<br>*Give P values as exact values whenever suitable.*
☒	☐	For Bayesian analysis, information on the choice of priors and Markov chain Monte Carlo settings
☒	☐	For hierarchical and complex designs, identification of the appropriate level for tests and full reporting of outcomes
☒	☐	Estimates of effect sizes (e.g. Cohen's *d*, Pearson's *r*), indicating how they were calculated

Our web collection on statistics for biologists contains articles on many of the points above.

Software and code

Policy information about availability of computer code

Data collection	The LARS1 3D structures was collected using PyMOL v2.4.0, Vienna-PTM server and Maestro software v12.3<br>For western blotting, images were collected with Gelcapture Microchemi v4.2(Protein Sample).<br>For OCR analysis, raw data were collected using XFe24 extracellular flux analyzer and WAVE software v2.6.0.31 (Agilent Technologies)<br>For circular dichroism analysis, raw data were collected using j-715 spectropolarimeter (jasco)<br>For ATP assay, raw data were collected using a plate reader (Infinite 200 Pro, Tecan).<br>For cell death, raw data were collected using Incucyte Zoom (Essen BioScience)<br>For leucylation, raw data were collected using liquid scintillation counter (HIDEX 300SL)
Data analysis	In general, results presented as mean ± SEM were collected and calculated using GraphPad Prism (v8.0)<br>For western blotting, The raw datasets were analyzed using Image J v1.51 and Multi gauge v3.0<br>For Immunofluorescence staining, The raw datasets were analyzed using Zen colocalization (Zen imaging software v7.0)<br>For LC-MS/MS analysis, raw data were analyzed using Scaffold Software (v4.0)<br>For cell death, raw data were analyzed using Incucyte Zoom 2018A (Essen BioScience)

For manuscripts utilizing custom algorithms or software that are central to the research but not yet described in published literature, software must be made available to editors and reviewers. We strongly encourage code deposition in a community repository (e.g. GitHub). See the Nature Portfolio guidelines for submitting code & software for further information.

March 2021

Data

Policy information about availability of data

All manuscripts must include a data availability statement. This statement should provide the following information, where applicable:

- Accession codes, unique identifiers, or web links for publicly available datasets
- A description of any restrictions on data availability
- For clinical datasets or third party data, please ensure that the statement adheres to our policy

The data that support this study are available from the corresponding authors upon reasonable request. The LARS1 3D structure referenced in this study are available from the PDB website. 6KQY [https://www.rcsb.org/structure/6kqy], 6KR7 [https://www.rcsb.org/structure/6KR7]. Source data are provided with this paper.

Field-specific reporting

Please select the one below that is the best fit for your research. If you are not sure, read the appropriate sections before making your selection.

☒ Life sciences ☐ Behavioural & social sciences ☐ Ecological, evolutionary & environmental sciences

For a reference copy of the document with all sections, see nature.com/documents/nr-reporting-summary-flat.pdf

Life sciences study design

All studies must disclose on these points even when the disclosure is negative.

Sample size	The sample size for each treatment group is detailed in the Figure Legends and Materials and Methods section. All experiments were performed in three independent repeats in every case to ensure reproducibility. For in vitro and in vivo experiments, sample size selection was based on the lab's previous experience.
Data exclusions	Failed immunoblots that could not be quantified because of low signal to noise ratios were excluded.
Replication	Each experiment involved n=3 independent experiments. For quantitative measurements, three or more independent experiments were carried out and statistical analysis performed. All attempts at replication were successful.
Randomization	For cell-based experiments Western blotting, cell types were known when prepare the samples or start to treat cells at the beginning of experiments. Randomization was performed on capturing regions of interest (ROIs) in fluorescence image analyses. In other experiments, rigorous and unbiased quantifications were performed using analyzer (Image J and Multi gauge).
Blinding	Blinding was not required for this study because there were no data exclusions and conclusions were made on objective quantitative analysis of data

Reporting for specific materials, systems and methods

We require information from authors about some types of materials, experimental systems and methods used in many studies. Here, indicate whether each material, system or method listed is relevant to your study. If you are not sure if a list item applies to your research, read the appropriate section before selecting a response.

Materials & experimental systems

n/a	Involved in the study	
☐	☒	Antibodies
☐	☒	Eukaryotic cell lines
☒	☐	Palaeontology and archaeology
☒	☐	Animals and other organisms
☒	☐	Human research participants
☒	☐	Clinical data
☒	☐	Dual use research of concern

Methods

n/a	Involved in the study	
☒	☐	ChIP-seq
☒	☐	Flow cytometry
☒	☐	MRI-based neuroimaging

Antibodies

Antibodies used	Rabbit polyclonal anti-phospho-p70 S6 kinase (T389)(WB dilution 1:1000, Cell signaling, #9205, RRID: AB_330944),<br>Rabbit polyclonal anti-p70 S6 kinase (WB dilution 1:1000, Cell signaling, #9202, RRID: AB_331676),<br>Rabbit monoclonal anti-phospho-4EBP1 (Thr37/46) (236B4) (WB dilution 1:1000, Cell signaling, #2855, RRID: AB_560835),<br>Rabbit monoclonal anti-4EBP1 (53H11) (WB dilution 1:1000, Cell signaling, #9644, RRID: AB_2097841),

March 2021

Rabbit monoclonal anti-RagB (D18F3) (WB dilution 1:1000, Cell signaling, #8150, RRID: AB_11178806),
Rabbit monoclonal anti-mTOR (7C10) (WB dilution 1:1000, Cell signaling, #2983, RRID: AB_2105622),
Rabbit monoclonal anti-Raptor (24C12) (WB dilution 1:1000, Cell signaling, #2280, RRID: AB_561245),
Rabbit monoclonal anti-Hexokinase II (C64G5) (WB dilution 1:1000, Cell signaling, #2867, RRID: AB_2232946),
Rabbit monoclonal anti-Aldolase A (D73H4) (WB dilution 1:1000, Cell signaling, #8060, RRID: AB_2797635),
Mouse monoclonal anti-DYKDDDDK Tag (Binds to same epitope as Sigma's Anti-FLAG® M2 Antibody) (9A3) (WB dilution 1:1000, Cell signaling, #8146, RRID: AB_10950495),
Rabbit monoclonal anti-phospho-AMPKα (T172), (40H9) (WB dilution 1:1000, Cell signaling, #2535, RRID:AB_331250),
Rabbit polyclonal anti-AMPKα (WB dilution 1:1000, Cell signaling, #2532, RRID:AB_330331),
Rabbit polyclonal anti-AMPKγ (WB dilution 1:1000, Cell signaling, #4187, RRID: AB_10695248),
Rabbit polyclonal anti-phospho-ULK1 (S757) (WB dilution 1:1000, Cell signaling, #6888, RRID: AB_10829226),
Rabbit monoclonal anti-phospho-Atg13 (S355), (D6J1W) (WB dilution 1:1000, Cell signaling, #26839, RRID: AB_2798932),
Rabbit monoclonal anti-Atg13 (D4P1K) (WB dilution 1:1000, Cell signaling, #13273, RRID: AB_2798169),
Rabbit monoclonal anti-phospho-Atg14 (S29), (D4B8M) (WB dilution 1:1000, Cell signaling, #92340, RRID: AB_2800182),
Rabbit monoclonal anti-Atg14 (D1A1N) (WB dilution 1:1000, Cell signaling, #96752, RRID: AB_2737056),
Rabbit polyclonal anti-LARS1 (WB dilution 1:1000, Cell signaling, #13868, RRID: AB_2798333),
Rabbit polyclonal anti-mTOR (WB dilution 1:1000, Cell signaling, #2972, RRID: AB_330978),
Rabbit monoclonal anti-ERp72 (D70D12) XP® (WB dilution 1:1000, Cell signaling, #5033, RRID: AB_10622112),
Rabbit polyclonal anti-RagD (WB dilution 1:1000, Bethyl Laboratories, #A304-301A, RRID: AB_2620497),
Rabbit polyclonal anti-LARS1 (WB dilution 1:1000, IF dilution 1:200, IP: 2ug/400ug protein, Bethyl Laboratories, #A304-315A, RRID: AB_2620511),
Mouse monoclonal anti-LAMP2 (H4B4) (WB dilution 1:1000, IF dilution 1:50, Santa cruz, #sc-18822, RRID: AB_626858),
Mouse monoclonal anti-ARF1 (ARFS 1A9/5) (WB dilution 1:1000, Santa cruz, #sc-53168, RRID: AB_2060825),
Mouse monoclonal anti-b-actin (C4) (WB dilution 1:1000, Santa cruz, #sc-47778, RRID: AB_626632),
Mouse monoclonal anti-c-Myc (9E10) (WB dilution 1:1000, IP: 2ug/300ug protein, Santa cruz #sc-40, RRID: AB_627268),
Mouse monoclonal anti-O-GlcNAc (RL2), (WB dilution 1:1000, Santa cruz, #sc-59624, RRID: AB_784963),
Mouse monoclonal anti-HA-Tag (F-7) (WB dilution 1:1000, Santa cruz, #sc-7392, RRID: AB_627809),
Rabbit polyclonal anti-ULK1 (H-240) (WB dilution 1:1000, Santa cruz, #sc-33182, RRID: AB_2214706),
Rabbit polyclonal anti-OGT1 / O-Linked N-Acetylglucosamine Transferase 1 (WB dilution 1:1000, IP: 2ug/400ug protein, abcam #ab96718, RRID: AB_10680015),
Rabbit polyclonal anti-phospho-PDCD4 (S67) (WB dilution 1:1000, abcam, #ab73343, RRID: AB_1524163),
Recombinant anti-PDCD4 (WB dilution 1:1000, abcam, #ab80590, RRID: AB_1603755),
Rabbit polyclonal anti-Aldolase B (WB dilution 1:8000, Proteintech, #18065-1-AP, RRID: AB_2273968),
Rabbit polyclonal anti-Aldolase C (WB dilution 1:5000, Proteintech, #14884-1-AP, RRID: AB_2226691),
Rabbit polyclonal anti-Sestrin 2 (WB dilution 1:2000, Proteintech, #10795-1-AP, RRID: AB_2185480),
Rabbit polyclonal anti-SESN1 (WB dilution 1:500, Proteintech, #21668-1-AP, RRID:AB_10793724),
Rabbit polyclonal anti-EPRS1 (WB dilution 1:5,000, Neomics, #NMS-01-0004,),
Goat anti-Rabbit IgG (H+L) Cross-Adsorbed Secondary Antibody, Alexa Fluor™ 488 (IF dilution 1:500 Invitrogen # A-11034, RRID: AB_2534069),
Goat anti-Mouse IgG (H+L) Cross-Adsorbed Secondary Antibody, Alexa Fluor™ 594 (IF dilution 1:500 Invitrogen #A-11005, RRID: AB_141359),
Goat anti-Mouse IgG (H+L) Secondary Antibody, HRP (WB dilution 1:10000 Invitrogen #31430, RRID: AB_228307),
and Goat anti-Rabbit IgG (H+L) Secondary Antibody, HRP (WB dilution 1:10000 Invitrogen #31460, RRID: AB_228341).

Validation

Antibodies used were commercially available and were validated in multiple previous studies. The following are the Research Resource Identifiers (RRIDs) from the Resource Identification Portal, supporting guidelines for Rigor and Transparency in scientific publications.

Rabbit polyclonal anti-phospho-p70 S6 kinase (T389)(RRID: AB_330944, https://www.cellsignal.com/products/primary-antibodies/phospho-p70-s6-kinase-thr389-antibody/9205)
Rabbit polyclonal anti-p70 S6 kinase (RRID: AB_331676, https://www.cellsignal.com/products/primary-antibodies/p70-s6-kinase-antibody/9202),
Rabbit monoclonal anti-phospho-4EBP1 (Thr37/46) (236B4) (RRID: AB_560835, https://www.cellsignal.com/products/primary-antibodies/phospho-4e-bp1-thr37-46-236b4-rabbit-mab/2855),
Rabbit monoclonal anti-4EBP1 (53H11) (RRID: AB_2097841, https://www.cellsignal.com/products/primary-antibodies/4e-bp1-53h11-rabbit-mab/9644),
Rabbit monoclonal anti-RagB (D18F3) (RRID: AB_11178806, https://www.cellsignal.com/products/primary-antibodies/ragb-d18f3-rabbit-mab/8150),
Rabbit monoclonal anti-mTOR (7C10) (RRID: AB_2105622, https://www.cellsignal.com/products/primary-antibodies/mtor-7c10-rabbit-mab/2983),
Rabbit monoclonal anti-Raptor (24C12) (RRID: AB_561245, https://www.cellsignal.com/products/primary-antibodies/raptor-24c12-rabbit-mab/2280),
Rabbit monoclonal anti-Hexokinase II (C64G5) (RRID: AB_2232946, https://www.cellsignal.com/products/primary-antibodies/hexokinase-ii-c64g5-rabbit-mab/2867),
Rabbit monoclonal anti-Aldolase A (D73H4) (RRID: AB_2797635, https://www.cellsignal.com/products/primary-antibodies/aldolase-a-d73h4-rabbit-mab/8060),
Mouse monoclonal anti-DYKDDDDK Tag (Binds to same epitope as Sigma's Anti-FLAG® M2 Antibody) (9A3) (RRID: AB_10950495, https://www.cellsignal.com/products/primary-antibodies/dykddddk-tag-9a3-mouse-mab-binds-to-same-epitope-as-sigma-s-anti-flag-m2-antibody/8146),
Rabbit monoclonal anti-phospho-AMPKα (T172), (40H9) (RRID:AB_331250, https://www.cellsignal.com/products/primary-antibodies/phospho-ampka-thr172-40h9-rabbit-mab/2535),
Rabbit polyclonal anti-AMPKα (RRID:AB_330331, https://www.cellsignal.com/products/primary-antibodies/ampka-antibody/2532),
Rabbit polyclonal anti-AMPKγ (RRID: AB_10695248, https://www.cellsignal.com/products/primary-antibodies/ampkg1-antibody/4187),
Rabbit polyclonal anti-phospho-ULK1 (S757) (RRID: AB_10829226, https://www.cellsignal.com/products/primary-antibodies/phospho-ulk1-ser757-antibody/6888),

Rabbit monoclonal anti-phospho-Atg13 (S355), (D6J1W) (RRID: AB_2798932, https://www.cellsignal.com/products/primary-antibodies/phospho-atg13-ser355-d6j1w-rabbit-mab/26839),
Rabbit monoclonal anti-Atg13 (D4P1K) (RRID: AB_2798169, https://www.cellsignal.com/products/primary-antibodies/atg13-d4p1k-rabbit-mab/13273),
Rabbit monoclonal anti-phospho-Atg14 (S29) (D4B8M) (RRID: AB_2800182, https://www.cellsignal.com/products/primary-antibodies/phospho-atg14-ser29-d4b8m-rabbit-mab/92340),
Rabbit monoclonal anti-Atg14 (D1A1N) (RRID: AB_2737056, https://www.cellsignal.com/products/primary-antibodies/atg14-d1a1n-rabbit-mab/96752),
Rabbit polyclonal anti-LARS1 (RRID: AB_2798333, https://www.cellsignal.com/products/primary-antibodies/lars-antibody/13868),
Rabbit polyclonal anti-mTOR (RRID: AB_330978, https://www.cellsignal.com/products/primary-antibodies/mtor-antibody/2972),
Rabbit monoclonal anti-ERp72 (D70D12) XP® (RRID: AB_10622112, https://www.cellsignal.com/products/primary-antibodies/erp72-d70d12-xp-rabbit-mab/5033),
Rabbit polyclonal anti-RagD (RRID: AB_2620497, https://www.thermofisher.com/antibody/product/RRAGD-Antibody-Polyclonal/A304-301A),
Rabbit polyclonal anti-LARS1 (RRID: AB_2620511, https://www.thermofisher.com/antibody/product/LARS-Antibody-Polyclonal/A304-315A),
Mouse monoclonal anti-LAMP2 (H4B4) (RRID: AB_626858, https://www.scbt.com/ko/p/lamp-2-antibody-h4b4?requestFrom=search),
Mouse monoclonal anti-ARF1 (ARFS 1A9/5) (RRID: AB_2060825, https://www.scbt.com/p/arf1-antibody-arfs-1a9-5?requestFrom=search),
Mouse monoclonal anti-b-actin (C4) (RRID: AB_626632, https://www.scbt.com/p/beta-actin-antibody-c4?requestFrom=search),
Mouse monoclonal anti-c-Myc (9E10) (RRID: AB_627268, https://www.scbt.com/p/c-myc-antibody-9e10?requestFrom=search),
Mouse monoclonal anti-O-GlcNAc (RL2), (RRID: AB_784963, https://www.citeab.com/antibodies/795670-sc-59624-o-glcnac-antibody-rl2),
Mouse monoclonal anti-HA-Tag (F-7) (RRID: AB_627809, https://www.scbt.com/p/ha-probe-antibody-f-7?requestFrom=search),
Rabbit polyclonal anti-ULK1 (H-240) (RRID: AB_2214706, https://www.scbt.com/p/ulk1-antibody-h-240),
Rabbit polyclonal anti-OGT1 / O-Linked N-Acetylglucosamine Transferase 1 (RRID: AB_10680015, https://www.abcam.com/ogt--o-linked-n-acetylglucosamine-transferase-antibody-ab96718.html),
Rabbit polyclonal anti-phospho-PDCD4 (S67) (RRID: AB_1524163, https://www.abcam.com/pdcd4-phospho-s67-antibody-ab73343.html),
Recombinant anti-PDCD4 (RRID: AB_1603755, https://www.abcam.com/pdcd4-antibody-epr3431-ab80590.html),
Rabbit polyclonal anti-Aldolase B (RRID: AB_2273968, https://www.ptglab.com/products/ALDOB-Antibody-18065-1-AP.htm),
Rabbit polyclonal anti-Aldolase C (RRID: AB_2226691, https://www.ptglab.com/products/ALDOC-Antibody-14884-1-AP.htm),
Rabbit polyclonal anti-Sestrin 2 (RRID: AB_2185480, https://www.ptglab.com/products/Sestrin2-Antibody-10795-1-AP.htm),
Rabbit polyclonal anti-SENS1 (RRID:AB_10793724, https://www.ptglab.com/products/SESN1-Antibody-21668-1-AP.htm)
Rabbit polyclonal anti-EPRS1 (WB dilution 1:5,000, Neomics, #NMS-01-0004, Glutamyl-Prolyl-tRNA Synthetase Regulates Epithelial Expression of Mesenchymal Markers and Extracellular Matrix Proteins: Implications for Idiopathic Pulmonary Fibrosis. Frontiers in pharmacology, 9, 1337. https://doi.org/10.3389/fphar.2018.01337),
Goat anti-Rabbit IgG (H+L) Cross-Adsorbed Secondary Antibody, Alexa Fluor™ 488 (RRID: AB_2534069, https://www.thermofisher.com/antibody/product/A-11034.html?ef_id=Cj0KCQjw06OTBhC_ARIsAAU1yOUXrIsE03a0wsdTHDKo3_usaG2XwAoJm16iC4fHp95OmD_hjgDOnxEaAt_tEALw_wcB:G:s&s_kwcid=AL!3652!3!516608152215!!!g!!&cid=bid_pca_aus_r01_co_cp1359_pjt0000_bid00000_0se_gaw_dy_pur_con&gclid=Cj0KCQjw06OTBhC_ARIsAAU1yOUXrIsE03a0wsdTHDKo3_usaG2XwAoJm16iC4fHp95OmD_hjgDOnxEaAt_tEALw_wcB),
Goat anti-Mouse IgG (H+L) Cross-Adsorbed Secondary Antibody, Alexa Fluor™ 594 (RRID: AB_141359, https://www.thermofisher.com/antibody/product/Goat-anti-Mouse-IgG-H-L-Cross-Adsorbed-Secondary-Antibody-Polyclonal/A-11005),
Goat anti-Mouse IgG (H+L) Secondary Antibody, HRP (RRID: AB_228307, https://www.thermofisher.com/antibody/product/Goat-anti-Mouse-IgG-H-L-Secondary-Antibody-Polyclonal/31430),
and Goat anti-Rabbit IgG (H+L) Secondary Antibody, HRP (RRID: AB_228341, https://www.thermofisher.com/antibody/product/Goat-anti-Rabbit-IgG-H-L-Secondary-Antibody-Polyclonal/31460).

Antibody validation for Immunofluorescence staining, immunoprecipitation, In vitro pulldown assay and In vitro GTPase assay were conducted based on previous experimental experience.

Eukaryotic cell lines

Policy information about cell lines

Cell line source(s)	SW620 (human, male) ATCC Cat#CCL-227 , RRID:CVCL_0547<br>SW480 (human, male) ATCC Cat#CCL-228 , RRID:CVCL_0546<br>PANC1 (human, male) ATCC Cat#CRL-1469 , RRID:CVCL_0480<br>A549 (human, male) ATCC Cat#CRL-185 , RRID:CVCL_0023<br>HeLa (human, female) ATCC Cat#CCL-2 , RRID:CVCL_0030<br>RD (human, female) ATCC Cat#CCL-136 , RRID:CVCL_1649<br>HEK293T (human, female) Korean Cell Line Bank Cat#21573, RRID:CVCL_0045<br>AMPKγ1 WT or AMPKγ1 KO 293A cell line was a kind gift of Prof. Hyun Woo Park (Yonsei University, Seoul, Korea)
Authentication	Cell lines were authenticated using morphology and carefully labeled and tracked in our lab. As stated in the methods, they were not used beyond 20 passages.
Mycoplasma contamination	Cells used for all experiments were mycoplasma negative.
Commonly misidentified lines<br>(See ICLAC register)	None of commonly misidentified cell lines has been used.
